# "Is Cybermedicine Killing You?" - A Response From the Authors of the Cochrane Review: Author's Reply (2)

**DOI:** 10.2196/jmir.7.4.e42

**Published:** 2005-07-28

**Authors:** Gunther Eysenbach

## Author's Response

We thank Murray and colleagues for adding their view to the "series of unfortunate events" outlined in the editorial [1] and in Rada's paper [2]. The editorial accurately describes the swift response of the authors in retracting the review within 13 days — unfortunately, at this time the cat was already out of the bag, and the media coverage had been substantial. We still think that, in order to reach Murray's aim of "ensuring that all relevant stakeholders were informed as quickly as possible" [3], it may not have been enough to "contact journalists who we knew to be writing articles about the original publication, but which had not yet been published," but also to contact those journalists who had already published stories, asking them to print corrections. We realize that this is — psychologically and practically — a difficult thing to do; however, it would have been the only way to ensure that the press coverage of the retraction matched the original coverage, which Murray and colleagues agree would have been better. The inaccuracies regarding the press release in the Rada paper are this author's responsibility and did not occur in the editorial. In fact, the complete University College London press release was published as a Multimedia Appendix to the editorial, including the retraction notice. While it is true that the press release mentions an interactive health communication application definition (which the editorial does not dispute), it is also a fact that this appears toward the end of the press release and was picked up by few journalists because the "Internet" is emphasized in the first paragraphs of the press release. This may have contributed to the confusion about the scope of the review.
